# SOX2OT lncRNA Inhibition Suppresses the Stemness Characteristics of Esophageal Tumorspheres

**DOI:** 10.3390/ncrna8060080

**Published:** 2022-11-28

**Authors:** Boshra Haghi, Marie Saghaeian Jazi, Ayyoob Khosravi, Seyyed Mehdi Jafari, Jahanbakhsh Asadi

**Affiliations:** 1 Metabolic Disorders Research Center, Golestan University of Medical Sciences, Gorgan 4934174515, Iran; 2 Stem Cell Research Center, Golestan University of Medical Sciences, Gorgan 4934174515, Iran; 3 Department of Molecular Medicine, Faculty of Advanced Medical Technologies Golestan, University of Medical Sciences, Gorgan 4934174516, Iran

**Keywords:** SOX2OT, docetaxel, tumorspheres, esophageal squamous cell carcinoma, chemoresistance, lncRNA

## Abstract

Background: SOX2OT is a novel cancer associated long non-coding RNA (LncRNA) with higher expression in variable tumor tissues, including esophageal squamous cell carcinoma (ESCC). It also plays an important function in embryonic neuronal development. Regarding its function in both stemness and carcinogenesis, here, we aimed to investigate its expression and function in tumorspheres of the esophagus using the RNAi method. Material & Methods: Two esophageal squamous cancer cells (ESCC): KYSE30 and YM1 cells were used for sphere enrichment. Cells were transfected with SOX2OT targeting and control siRNA. The size and the number of spheres were measured using light microscopy. Gene expression of the pluripotency genes was measured by qRT-PCR and docetaxel chemoresistance was assessed by MTS viability assay. Results: Our findings showed that ESCC tumorspheres overexpress SOX2OT gene along with other stemness genes (SOX2, OCT4A, and Nanog) compared to their original cancer cells. RNAi experiments indicated that SOX2OT knockdown can suppress the stemness-related gene expression, sphere formation ability (both size and number), and docetaxel resistance as three of the main cancer stem cell characteristics of tumorspheres. Conclusion: Altogether our results showed the regulatory role of SOX2OT in pluripotency and stemness in ESCC tumorspheres. Our results suggest a potential application of SOX2OT inhibition in combination with docetaxel for ESCC inhibition in vitro.

## 1. Introduction

Esophageal cancer is the eighth most common malignant tumor and sixth largest cause of cancer-related mortality worldwide with a poor prognosis [1,2,3]. Esophageal squamous cell carcinoma (ESCC) is one of the two most frequent and predominant histopathological types of esophagus carcinoma worldwide with a high prevalence in the northeast of Iran [4,5,6]. Various environmental and genetic factors are involved in the ESCC oncogenesis and progression [4,7]. Despite recent advances in cancer diagnosis and treatment, the recurrence and mortality rate of ESCC remains considerably high [8]. Chemotherapeutic strategies using docetaxel from the taxane family member are commonly used alone or in combination for cancer treatment. Docetaxel leads to cancer cell cycle arrest and apoptosis through tubulin B subunit binding and microtubule disruption [9]. Cancer cell drug resistance is a main concern in treatment outcomes. The variable molecular mechanisms may account for docetaxel resistance [10,11] including over-expression of multi-drug resistance gene encoding efflux pump, B tubulin, autophagy modulators, and mutation in pro-apoptotic genes. However, a clear molecular mechanism underlying docetaxel resistance is not defined yet [12,13].

Cancer stem cells (CSCs) are characterized as tumor-initiating cells with high proliferation potency [14] and are the cancer cell sub-population mainly responsible for chemotherapy resistance and tumor recurrences [15]. High expression of the ABC transporter and stemness genes in CSCs consequently can lead to failure of chemotherapy [16,17,18,19,20]. One of the master modulators of pluripotency in cancer stem cells, SOX2 is located in an intronic region of SOX2OT long non-coding RNA. SOX2OT is mapped on a highly conserved region in chromosome 3q26.3 in humans [21,22] and has various cellular functions in embryogenesis and neuronal development [23], cancer cell proliferation [24,25], metastasis [26], and drug resistance [27,28]. Previous studies support overexpression of SOX2OT in tumor tissues of the breast [29], esophageal [30], lung [24,31], and hepatocellular [26]. However, the exact molecular mechanism of SOX2OT function appeals to more investigation. Overexpression of SOX2 has been used as well-known pluripotency associated marker for CSC isolation [21]. There are several studies illustrating the important role of lncRNA in the progression of cancers through the regulation of CSCs, tumorspheres, and self-renewal [32]. Regarding the reported association between the SOX2 and SOX2OT, we aimed to investigate the expression and function of SOX2OT as a lncRNA in tumorspheres derived from esophageal cancer cells (YM1, KYSE30).

## 2. Results

### 2.1. The YM1- and KYSE30-Derived Spheres Represent Cancer Stem-like Characteristics

**Figure 1:**
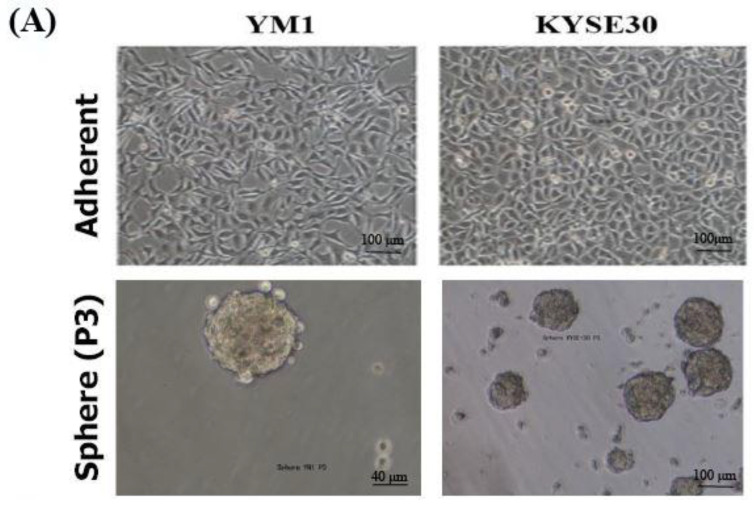

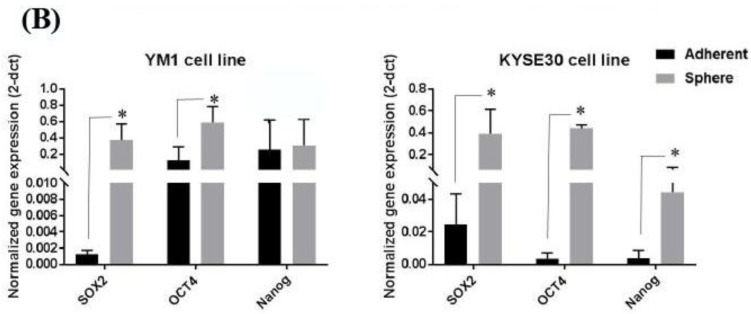
Stemness characterization of the YM1- and KYSE30-derived spheres. (**A**) shows the adherent form of the original cells and the tumorspheres in passage 3. (**B**) shows increased gene expression of the pluripotency regulators (SOX2, OCT4A, and Nanog) in spheres of compared to the adherent cells, measured by qRT-PCR. * *p*-value < 0.05.

**Figure 2:**
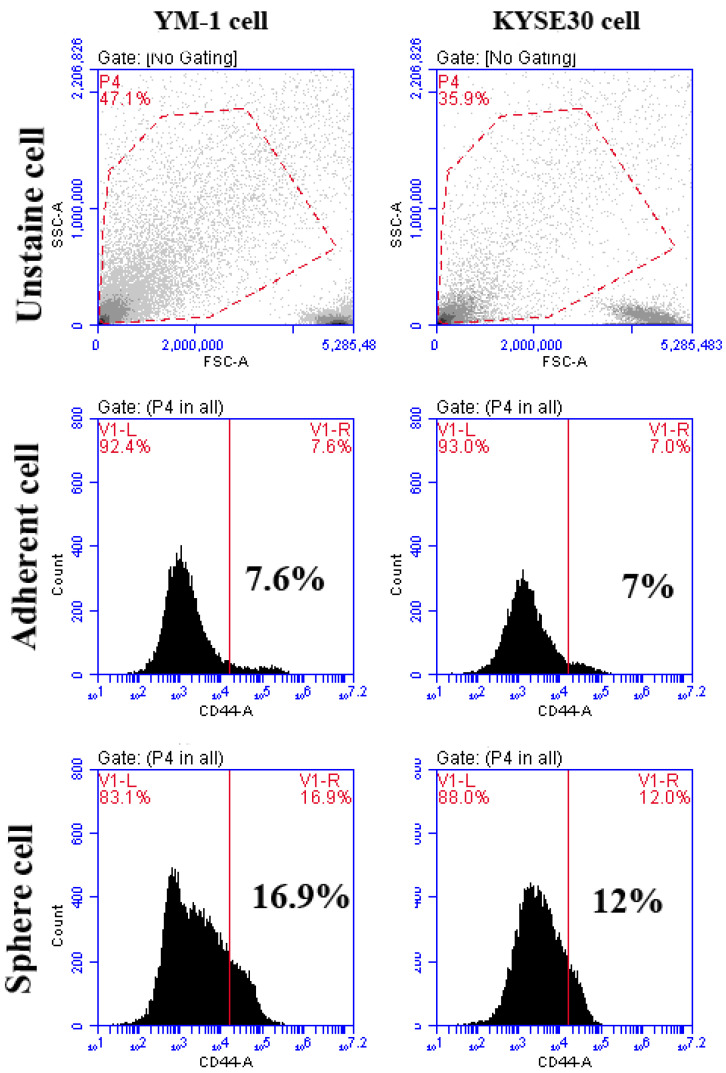
The flowcytometry analysis of CD44 cancer stem cell marker in adherent and tumorspheres. The percentages of CD44 positive cells are shown in the graphs. The unstained cell was used for background subtraction.

To ensure the stemness characteristics, the YM-1-derived spheres (YM1-S) and KYSE30-derived spheres (KYSE30-S) both in passage three were compared to their original attached cells (YM1 and KYSE30). As shown in Figure 1A, both cell lines made distinguished well-shaped spheres in passage 3. Further gene expression analysis of the spheres revealed statistically significant over-expression of the master regulators of the pluripotency genes (SOX2, OCT4A). The over-expression of Nanog was statistically significant in KYSE30-S cell line (Figure 1B). The sphere cells characterized by pluripotency gene upregulation and anchorage independent growth were confirmed as a side population with stemness features. Importantly, the expression of the CD44 as one of the gastrointestinal cancer stem cell markers was upregulated in tumorspheres of passage 3 (Figure 2). Indeed, the tumorsphere of both cell lines were more resistant to docetaxel (IC50) treatment, showing increased viability to about 75% for YM1-S and 85% for KYSE30-S cells, highlighting their stemness properties (data presented in following sections). Moreover, the tumorigenicity of the tumorspheres derived by this method was confirmed in our lab in previous studies [33,34].

### 2.2. Esophageal Tumorspheres Overexpress SOX2OT lncRNA

**Figure 3:**
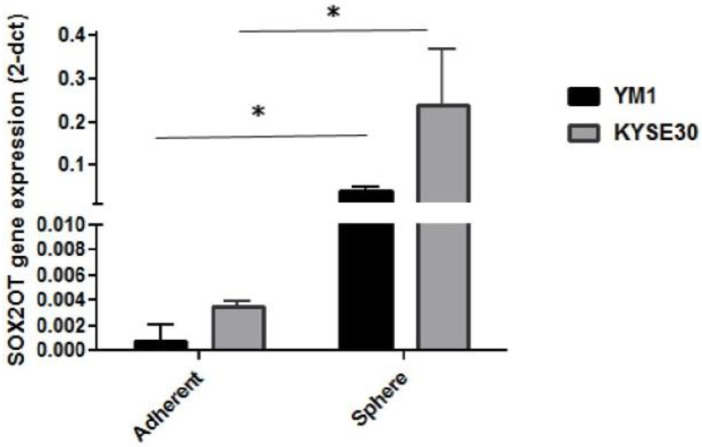
Overexpression of SOX2OT in tumorspheres of esophageal cancer cells (YM1 and KYSE30) * *p*-value < 0.05. bars represent mean ± SE.

To study the association of the SOX2OT lncRNA in ESCC tumorspheres, primarily, the expression changes of the SOX2OT were assessed in the tumorspheres of both cell lines. As it is shown in Figure 3, interestingly, both YM1 and KYSE30 spheres expressed a significantly higher expression of the SOX2OT. Upregulation of the SOX2OT in YM1-derived tumorspheres was higher than the KYSE30, which can be explained with the lower basic expression of the SOX2OT in the YM1-adherent cell compared to the KYSE30-adherent cell. These findings indicate the importance of SOX2OT lncRNA in the development of the tumorspheres in esophageal carcinoma.

### 2.3. SOX2OT Knockdown in Tumorspheres of ESCC

**Figure 4:**
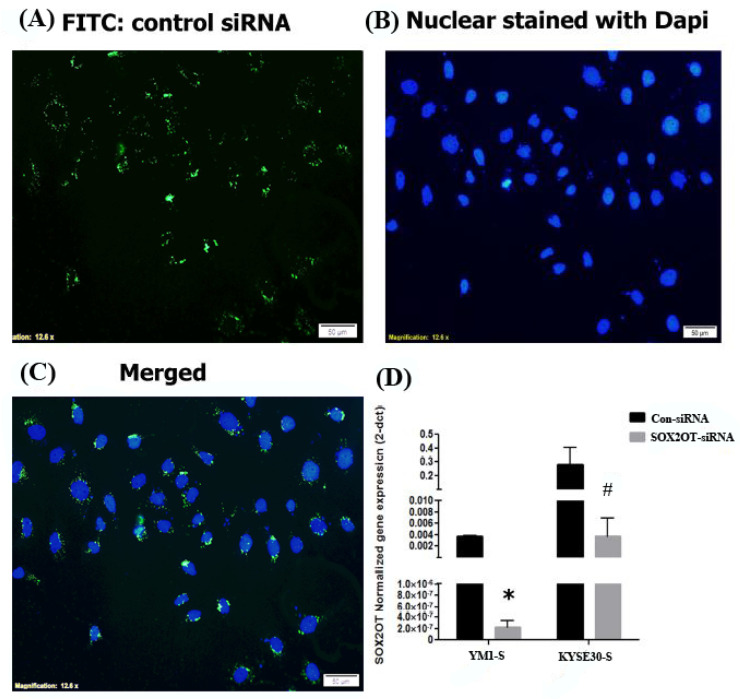
SOX2OT knockdown in ESCC cells. Fluorescence imaging of the FITC-scrambled siRNA transfected ESCCs. The transfected siRNA is shown in green (**A**) and nuclear is stained with dapi in blue (**B**) color. The merged image is shown in (**C**). The quantitative gene expression assessment demonstrated significant downregulation of the SOX2OT in siRNA-transfected YM1-S and KYSE30-S tumorspheres in comparison to the control scrambled siRNA-transfected tumorspheres (**D**). YM1-S: YM1 sphere, KYSE30-S: KYSE30 sphere, * *p*-value = 0.017 and # *p*-value = 0.05. bars represent mean ± SE.

For more investigation, SOX2OT siRNA was used to study its function in sphere formation ability and stemness features. Both ESCC cells were treated with control scrambled or SOX2OT targeting siRNA. To ensure siRNA delivery, transfection efficiency was evaluated approximately to >90% with fluorescence imaging of FITC conjugated scrambled siRNA (Figure 4A–C). Then, decreased expression of the SOX2OT was confirmed in siRNA-transfected tumorspheres of each cell line using qRT-PCR experiment. As shown in Figure 4D, the expression of SOX2OT in the tumorspheres of both YM1 and KYSE30 was significantly knocked down in the siRNA-transfected cells.

### 2.4. SOX2OT Downregulation Inhibits Stemness and Sphere Formation Ability of the ESCC Tumorspheres

**Figure 5:**
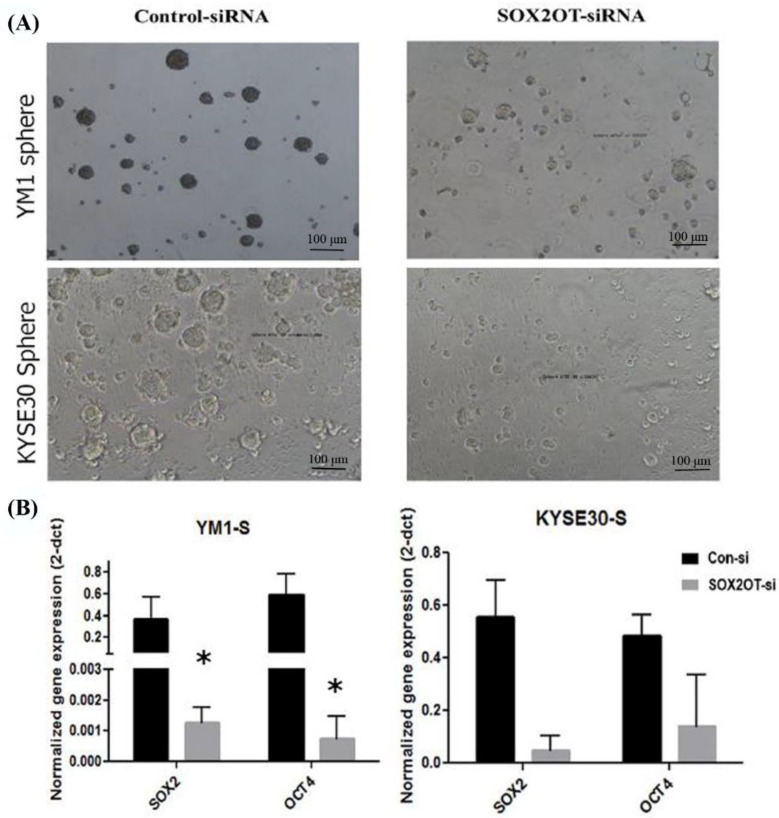
Downregulation of SOX2OT in YM1 and KYSE30 tumorspheres. (**A**) shows the phenotype of the tumorspheres of each cell line after SOX2OT siRNA or control non-target siRNA transfection. (**B**) shows the decreased expression of pluripotency genes in SOX2OT knocked down tumorspheres. YM1: * *p*-value = 0.05 (SOX2, OCT4A) and KYSE30: *p*-value = 0.064 SOX2, *p*-value = 0.083 OCT4A and bars represent mean ± SE.

For functional evaluation of the SOX2OT inhibition in tumorspheres development, the stemness characterization experiments were repeated after SOX2OT knockdown in sphere cells. As expected, our findings showed that SOX2OT knockdown in YM1 and KYSE30 spheres disrupt the tumorsphere integrity, changing the shape to smaller spheres with distorted borders and consequently decreasing the tumorsphere ability (Figure 5A). Moreover, the expression level of the pluripotency genes (SOX2, OCT4A) was downregulated in tumorspheres after SOX2OT knockdown. The concordant downregulation of the SOX2 and OCT4A was significant in SOX2OT knocked down YM1 tumorspheres; however, in KYSE30 tumorspheres after SOX2OT inhibition, we found non-significant decreased expression of SOX2 (*p* value = 0.064) and OCT4A (*p* value = 0.083) (Figure 5B).

**Figure 6:**
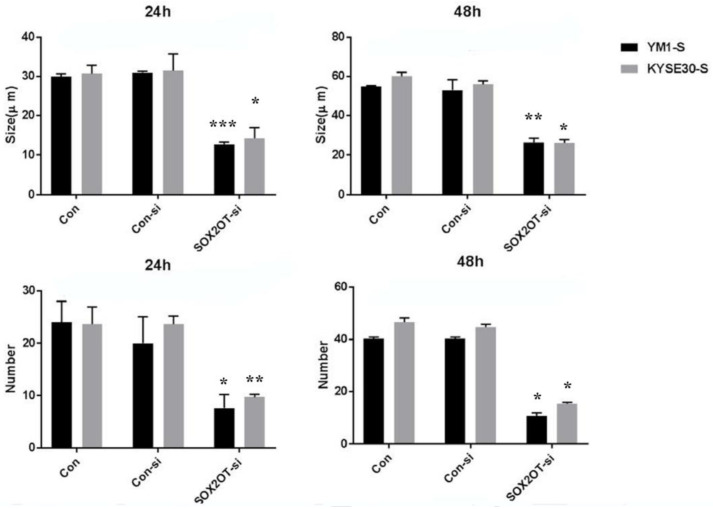
The sphere formation capability of ESCC tumorspheres after SOX2OT knockdown. The comparison was carried out between SOX2OT knocked down (SOX2OT-si) and control siRNA transfected (Con-si) tumorspheres of each cell line. Both sphere diameter and count were measured in at least five fields with light microscopy for 24 and 48 h after transfection. * *p*-value ≤ 0.05 and ** *p*-value < 0.003 and *** *p*-value = 0.0001 vs. control.

The sphere formation capability of the SOX2OT knocked down cells was also measured regarding the number and the size of the spheres. As is shown in Figure 6, the SOX2OT knocked down tumorspheres from both YM1 and KYSE30 made fewer and smaller spheres compared to the control non-target siRNA-transfected tumorspheres. The diameter of the spheres declined approximately to half of its size following SOX2OT siRNA transfection in both time points 24 and 48 h (for YM1, *p* value = 0.0001 and *p* value = 0.003; for KYSE30, *p* value = 0.05 and *p* value = 0.04 for each time point). Moreover, the count of spheres/Petri dish was decreased significantly following 24 and 48 h after SOX2OT knockdown in both cell lines (for YM1, *p* value = 0.05 and *p* value = 0.04; for KYSE30, *p* value = 0.001 and *p* value = 0.04 for each time point).

### 2.5. SOX2OT Knockdown Enhances Docetaxel Toxicity in ESCC Tumorspheres

**Figure 7:**
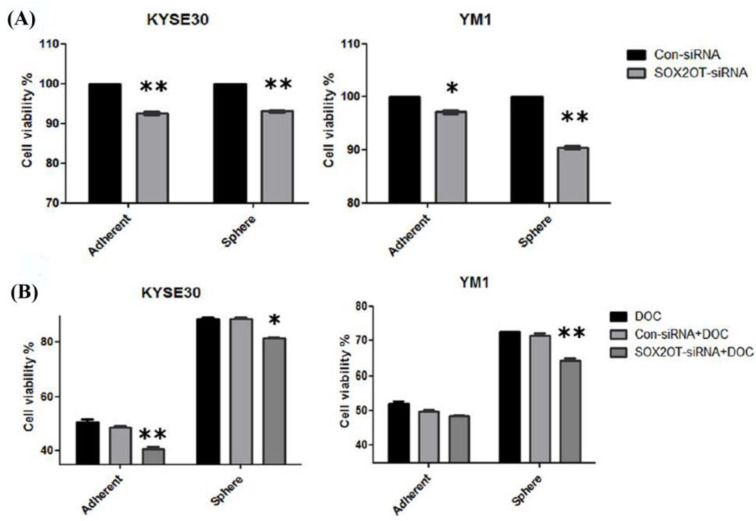
SOX2OT knockdown decreases the viability alone or in combination with docetaxel. The cytotoxicity of SOX2OT knockdown has been shown for both cell line adherents and spheres (**A**). The chemotoxicity of docetaxel (IC50) alone or in combination with SOX2OT-siRNA has been shown for both cell lines adherent and sphere cells (**B**). Doc: docetaxel. The two-way ANOVA test with Bonferroni comparison has been calculated by graph pad. * *p*-value < 0.01 and ** *p*-value < 0.001. Each Bar represents mean ± SE.

One of the greatest concerns in cancer recurrence is chemoresistance associated with CSCs. Our findings indicated that SOX2OT knockdown alone can have a minor significant toxicity, resulting in a decrease in the percentage of viable cells of both adherent and tumorspheres for both ESCC cell lines (~10%) when compared to control siRNA-transfected cells (Figure 7A). As one of the CSCs characteristics, we found both of the enriched tumorspheres populations to be more resistant to docetaxel (IC50 for each cell line) with ~25% toxicity for YM1 tumorspheres and ~15% for KYSE30 tumorspheres in comparison to the adherent cells (~50% toxicity) (Figure 7B, DOC representing bars). To address the association of SOX2OT in tumorspheres resistance to chemotherapy, cell viability was measured again in SOX2OT-siRNA treated cells in combination with docetaxel treatment (calculated IC50 for each cell line). Interestingly, we observed that RNAi inhibition of SOX2OT can significantly enhance the toxicity of the docetaxel in KYSE30-adherent and sphere cells, but only in YM1 sphere cells when compared to docetaxel.

This suggests that, by using SOX2OT inhibition, it is possible to overcome the chemoresistance to docetaxel in both esophageal cancer stem-like (tumorsphere) cells to some extent. The final chemotoxicity of docetaxel + SOX2OT-siRNA in combination achieved ~30% for YM1 tumorspheres and ~22% for KYSE30 tumorspheres (Figure 7B).

## 3. Discussion

SOX2 overlapping transcript is a lncRNA associated with cancer progression and embryonic stem cell development. Different studies have reported upregulation of the SOX2OT in solid tumors including breast [29], lung [24,31], ovarian [35], hepatocellular [26], colorectal [25], and esophageal [30,36] carcinoma. The CSCs have been identified as tumor initiation sub-population cells [37] in esophageal squamous cell carcinoma, related to chemoresistance and poor survival of the patients [38]. It has been reported that SOX2OT inhibition can decrease the colony formation ability or proliferation of ovarian [35], lung [24,31], breast [29], hepatocellular [26], and colorectal [25] cancer cells. Additionally, in our experiments, SOX2OT inhibition by siRNA decreased the ESCC-adherent cells (YM1 and KYSE30) viability. Wu et al. illustrated that SOX2OT ectopic expression can promote esophageal cancer cell lines (KYSE150, KYSE450) in vitro even in the presence of Cisplatin [30].

Here, in the current study, we showed significant upregulation of SOX2OT in tumorspheres of two different ESCC cells: YM1 and KYSE30. We found that SOX2OT inhibition decreased the stemness associated genes including the SOX2, OCT4A, and Nanog. In accordance with our study, recently it has been reported that SOX2OT expression is higher in glioblastoma stem cells compared to the original cancer cell lines (U87 and U251) [39]. Moreover, in another study, it was reported that knockdown of SOX2OT suppressed the stemness phenotype of bladder cancer stem cells (BCSC), indicating its important role in the regulation of bladder cancer stem cells [40]. Considering the overlapping location of SOX2 and SOX2OT, it was postulated that SOX2OT may contribute to SOX2 control [41]. Some evidence suggests that it can regulate SOX2 (stem cell regulator) maintenance and expression concordantly [29]; however, recently it has been reported that SOX2OT inversely correlates with SOX2, repressing its transcription in mice neuronal differentiation processes [42,43]. The underlying mechanism that SOX2OT can regulate SOX2 is not clearly determined yet, but studies illustrated different mechanisms. For example, Knauss et al. showed that SOX2OT can regulate sox2 expression negatively by a mechanism dependent to YY1, a transcriptional regulator, which can bind to CpG islands in the Sox2 locus [42]. On the other hand, Zhan et al. reported that SOX2OT can positively regulate SOX2 expression by sponging miR-200c non-coding RNA in bladder cancer stem cell [40].

In the current study we observed the downregulation of pluripotency genes including SOX2 upon SOX2OT knockdown, proposing a direct correlation between SOX2OT with the stemness genes (SOX2, OCT4A, and Nanog), particularly in ESCC tumorspheres. Previous reports support the regulated expression of SOX2OT in embryonic stem cell regulation and vertebrate development [44]. Interestingly, Su et al. showed that SOX2OT variant 4 inhibition using shRNA can decrease proliferation, migration, and invasion of the U87 and U251 glioblastoma stem cells [39]. Our results indicated that total SOX2OT inhibition with siRNA can limit the sphere formation ability and viability of ESCC (YM1, KYSE30) tumorspheres. Wang et al. showed that CD133+ and CD44+ subpopulation of Osteosarcoma cell lines considerably overexpresses SOX2OT (variant 7) when compared to control. Additionally, ectopic upregulation of SOX2OT (through lentivirus) in osteosarcoma cancer stem cells functionally increased sphere formation potential [27] which highlights its possible function in CSCs. One of the main concerns in cancer therapy is to overcome the chemoresistance of cancer cells especially related to CSCs. There is some evidence demonstrating SOX2OT association with chemoresistance of cancer cells. Ectopic overexpression of SOX2OT in breast cancer cell line MDAMB231 resulted in the decreased mitotic arrest in paclitaxel treated cells [29]. Our findings illustrated that SOX2OT RNAi inhibition can synergize the toxicity of docetaxel—another taxane chemo drug—in ESCC tumorspheres. There is some supporting evidence highlighting SOX2OT association with drug resistance to other chemotherapy agents with a different mechanism. It has been reported that SOX2OT inhibition can restrict the bladder cancer stem cell (CD44+, ALDH1+) self-renewal and cisplatin resistance by SOX2 regulation [28]. Wang et al. showed that EGCG (polyphenol derived from green tea), which targets SOX2OT, can synergize doxorubicin toxicity in osteosarcoma cells by autophagy and stemness inhibition [27]. Altogether, it may suggest a promising application of SOX2OT targeting in esophagus cancer therapy, particularly for tumorspheres inhibition. However, our findings are limited to in vitro carried out at cell culture level and it is necessary to be confirmed in animal models with in vivo condition in future studies.

## 4. Materials and Methods

### 4.1. Cancer Cell Culture

Two human esophageal squamous cancer cell lines were used: KYSE30 (ordered from Pasteur Institute, Tehran, Iran) and YM1 (previously established in our lab) [45] YM1 cells were cultured in DMEM/F12 Glutamax (low Glucose) and KYSE30 was maintained in RPMI 1640 medium, both supplemented with FBS 10% (cat number: BI-1201, Bio idea company, Iran) and Pen/Strep 1% (cat number: BI-1203, Bio idea company, Iran) in 5% CO2 incubator at 37 °C temperature. The medium was refreshed every 3 days during the experiments. For tumorspheres enrichment, the adherent cells were passaged in DMEM supplemented with FBS 5% for two passages. Then, 105 cells were cultured in a low attach Petri dish 6 cm (labtron, Iran) in DMEM containing growth factors EGF (20 ng/mL-Royan-Iran), bFGF (20 ng/mL-Royan-Iran), and B27 (2%, without vitamin A- Invitrogen). The cell culture medium was refreshed every two days up to the third passage which was used for the next experiments. To confirm the tumorspheres enrichment, expression of the pluripotency associated genes (SOX2, OCT4A, and Nanog) and docetaxel toxicity were assessed as explained below.

### 4.2. Cell Viability Assay

Cells were treated with different concentrations of the Docetaxel in a medium containing FBS 5% for 72 h (for YM1) and 48 h (for KYSE30) regarding the doubling time of the cells. Since the toxicity of the Docetaxel was dependent on the mitotic cell cycle, the FBS was not omitted during the viability and toxicity assays. The IC50 was calculated at 453 nM (R2 = 0.9804) for YM1 and 222 nM (R^2^ = 0.9022) for KYSE30 using the MTT viability assay according to the manufacturer’s protocol. For chemoresistance assessment, either transfected/non-transfected sphere or adherent forms of YM1 or KYSE30 cells were treated with IC50 and then the cell viability was measured using MTS assay following the producer’s suggested protocol.

### 4.3. Cell Transfection

Briefly, 6 × 10^4^ adherent cells were cultured in 24-well plates, and then cells were transfected after 24 h with SOX2OT-targeting siRNA 100 nM (from Dharmacon, Lafayette, CO, USA) or control non-target siRNA labeled with FITC 25 nM (Santa-cruz, Santa Cruz, CA, USA) using Viromer blue transfection reagent (Lipocalyx, GmbH, Halle, Germany) according to the manufacturer’s protocol. To ensure efficient knockdown, Lincode Human SOX2-OT siRNA—SMARTpool, (Catalog ID:R-188238-00-0010) was used, which is a mixture of 4 siRNA provided as a single reagent, thus targeting multiple regions of the lncRNA to improve the likelihood of RNAi-mediated degradation. Transfection medium was replaced following 6 h with fresh medium or docetaxel-containing medium in the case of cell toxicity assay. Approximately 6 × 10^4^ Sphere cells were transfected similarly but in the non-treated 24-well plates. The transfection efficacy was calculated using fluorescence microscopy of the control siRNA-FITC-transfected YM1 cells. The nuclear stain Dapi was used for nuclear visualization and the images were analyzed using ImageJ software (V1.42). The efficiency of transfection was formulated as the percentage of the transfected cells (green, FITC + cell count) to the nucleus of total cells (blue, Dapi + cell count) which were counted using a fluorescent microscopy instrument (Nikon, Tokyo, Japan).

### 4.4. Sphere Formation Assay

To compare the sphere formation capacity of the SOX2OT knocked down cells at two different time points after transfection (24 h and 48 h), the control siRNA and SOX2OT siRNA-transfected sphere cells were monitored with light microscopy by a blind observer. The number and size of the spheres were recorded in at least five fields of each sample. The sphere size stands for the sphere diameter in micrometer, which was measured using the Nicon microscope tool (Nikon, Tokyo, Japan).

### 4.5. Gene Expression Assay

Triplicates samples of the total RNAs were extracted from both attached and spheres of the YM1 and KYSE30 cell lines using TRIzol reagent (Ambion, TX, USA), according to the suggested protocol by the producer. A total amount of 1 μg DNAseI (Yekta Tajhiz Azma, Tehran, Iran) treated RNA was used for cDNA synthesis (Yekta Tajhiz Azma, Tehran, Iran) using the manufacturer’s procedure. The gene expression was measured using qRT-PCR method by Amplicon SYBR green master mix using specific primers for the target genes (Table 1). The target genes were amplified in the following condition: incubation at 95 °C for 10 min, 40 cycles of the 95 °C for 10 s, annealing at 61 °C for 20 s, and the extension at 72 °C for 40 s. To confirm the specificity of amplification, the melting curve was analyzed. For melt curve analysis, briefly the fluorescent was captured during temperature increase from 65 to 90 °C and then the Tm (melting temperature) of each PCR product was evaluated. The GAPDH gene expression was used as a housekeeping gene and the 2-dct method was used for the gene expression normalization. The qRT-PCR experiments were carried out by using the light cycler (Roche, Basel, Switzerland).

### 4.6. Statistical Analysis

All measurements and treatment were repeated at least in three replicates. The SPSS 16.0 and Graph pad Prism v 5 were used for statistical analyses of the data considering *p* values < 0.05 to be significant. To compare the difference of the measured variables, the Mann–Whitney U or T-test was used for comparison of two groups or two-way ANOVA test (Bonferroni multiple comparisons) was used for comparison of mor than two groups.

## 5. Conclusions

Altogether, our findings indicate significant SOX2OT lncRNA upregulation in tumorspheres of ESCC (YM1 and KYSE30) associated with pluripotency genes (SOX2, OCT4A, Nanog) expression, sphere formation capability, and docetaxel chemoresistance. Our results suggest promising benefits of SOX2OT knockdown in combination with docetaxel for ESCC tumorsphere inhibition and esophageal cancer therapy.

## Figures and Tables

**Table 1:** Specific primers sequence of the target genes.

Gene Symbol	Primer Sequence
*SOX2OT*	F: GGCTGGGAAGGACAGTTCG, R: AGATGATCTTGCCAGGCGATC
*SOX2*	F: TACAGCATGTCCTACTCGCAG, R: GAGGAAGAGGTAACCACAGGG
*OCT4A*	F: GTCGAGAGCAACTCCGATG, R:TGCTCCAGCTTCTCCTTCTC
*Nanog*	F: ATTCAGGACAGCCCTGATTCTTC, R: TTTTTGCGACACTCTTCTCTGC
*GAPDH*	F: AAGGTGAAGGTCGGAGTCAA, R: AATGAAGGGGTCATTGATGG

## Data Availability

All data is included in the manuscript.
